# Dosis de radiación permitida en pacientes odontológicos. Una revisión

**DOI:** 10.21142/2523-2754-1101-2023-144

**Published:** 2023-03-26

**Authors:** Omar Pinto Nicodemo, Gustavo Adolfo Fiori-Chíncaro, Ana María Agudelo-Botero, Jhoana M Llaguno-Rubio, Rosaura García Díaz

**Affiliations:** 1 Carrera de Estomatologia, Universidad Mayor, Real y Pontificia de San Francisco Xavier de Chuquisaca. Sucre, Bolivia. omarpinto_nicodemo@hotmail.com Universidad Mayor de San Francisco Xavier Carrera de Estomatologia Universidad Mayor, Real y Pontificia de San Francisco Xavier de Chuquisaca Sucre Bolivia omarpinto_nicodemo@hotmail.com; 2 Division de Radiologia Bucal y Maxilofacial, Instituto Latinoamericano de Altos Estudios en Estomatologia. Lima, Peru. gfiori@ilaeperu.com, academico@ilaeperu.com Division de Radiologia Bucal y Maxilofacial Instituto Latinoamericano de Altos Estudios en Estomatologia Lima Peru gfiori@ilaeperu.com academico@ilaeperu.com; 3 Facultad de Estomatologia, Universidad Autonoma de Manizales. Manizales, Colombia. ortoamariabotero@gmail.com Universidad Autonoma de Manizales Facultad de Estomatologia Universidad Autonoma de Manizales Manizales Colombia ortoamariabotero@gmail.com; 4 Facultad de Estomatologia, Universidad de Guadalajara. Guadalajara, Mexico. dentys_gard@hotmail.com Universidad de Guadalajara Facultad de Estomatologia Universidad de Guadalajara Guadalajara Mexico dentys_gard@hotmail.com

**Keywords:** consultorios odontológicos, dosis de radiación, rayos x, atención ambulatoria, dental offices, radiation dosage, x-ray, ambulatory care

## Abstract

En la práctica odontológica, el uso de la imagenología se ha incrementado con los años, lo cual ha generado un aumento en la dosis de radiación para el paciente odontológico. Un factor en discusión es la cantidad de dosis usadas en pacientes (hombres, mujeres y niños) en diferentes etapas de su vida, por la evidencia científica de los efectos nocivos de las radiaciones ionizantes. Esta revisión de la literatura analizó las características de las radiaciones y sus efectos en relación con las dosis administradas, así como sus equivalencias en la práctica odontológica, en las radiografías periapicales panorámicas y tomografías de haz cónico. Se realizó una búsqueda de la literatura en las principales fuentes de información, como Medline (vía PubMed), Elsevier, SciELO y LILACS, usando los términos de búsqueda con una limitación de fecha de los últimos 10 años. Los artículos seleccionados incluyeron información referente a las palabras clave utilizadas: “consultorios odontológicos”, “dosis de radiación”, “rayos X” y “atención ambulatoria”.

## Abstract (en)

In dental practice, the use of imaging has increased over the years, generating an increase in the radiation dose for the dental patient. One factor under discussion is the amount of dose used in patients, men, women and children, at different stages of their life, due to the scientific evidence of the harmful effects of ionizing radiation. This review of the literature analyzed the characteristics of radiation and its effects in relation to the doses administered, and its equivalences in dental practice in panoramic periapical radiographs and cone beam tomographies. A search of the literature was carried out in the main information sources such as Medline (via PubMed), SEVIER, SCIELO, and LILACS, using the search terms with a date limitation of the last 10 years. The selected articles had to include information regarding the keywords used, including "Dental Offices, Radiation Dose, X-Rays, Ambulatory Care."

## INTRODUCCIÓN

En la práctica odontológica, el uso de imagenología se ha incrementado con los años, y constituye uno de los pilares fundamentales del diagnóstico de rutina, hecho que ha generado un incremento en la dosis de radiación para el paciente odontológico ^1^.

Uno de los factores que se ha puesto siempre en discusión es la cantidad de dosis utilizada en pacientes, en diferentes etapas de su vida, por la evidencia científica de los efectos nocivos de las radiaciones ionizantes ^2^.

En la actualidad, el uso de los diferentes tipos de modalidades imagenológicas, como las radiografías y tomografías de haz cónico (TCHC), en los diversos procedimientos dentales se ha incrementado en gran medida ^3^^-^^5^, lo que ha provocado un aumento en la exposición del paciente a las dosis de radiaciones ionizantes e incrementado el riesgo de los efectos nocivos, estocásticos y determinísticos ^6^.

Las magnitudes de estas exposiciones dependen principalmente de los parámetros utilizados en radiología ^7^^,^^8^. Los niveles de dosis y sus efectos nocivos, la “cantidad de energía absorbida por una unidad de masa en una región de interés” ^2^^,^^9^, han sido modificados y regulados a través del tiempo, con el respaldo de una continua investigación científica en el mundo ^3^^,^^4^^,^^10^.

La optimización de las dosis es el principio fundamental de la radioprotección, tanto en los pacientes como en el personal de asistencia médica-odontológica. “Mantener las dosis tan bajas como sea razonablemente posible” (principios ALARA-ALADA) es el objetivo fundamental de la Comisión Internacional de Protección Radiológica 1990-2007 ^11^^,^^12^.

La justificación, la optimización y el límite de dosis no son más que restricciones para disminuir el riesgo a los rayos ionizantes en las diferentes instancias diagnósticas, considerando las dosis de radiación utilizadas y las dosis acumuladas a través del tiempo por la frecuencia de exposición ^13^^,^^14^. 

En este sentido, esta revisión sistemática de la literatura tuvo el objetivo de analizar y describir las características de las radiaciones y sus efectos en relación con las dosis administradas, así como sus equivalencias en la práctica odontológica, en las radiografías periapicales panorámicas y las tomografías de haz cónico. Para ello, se tuvieron en cuenta las diferentes fuentes de emisión radiológica a las que están expuestos los pacientes y el personal de salud.

## MATERIALES Y MÉTODOS

Se realizó una búsqueda de la literatura en las principales fuentes de información, incluyendo Medline (vía PubMed), Elsevier, SciELO y LILACS, usando los términos de búsqueda especializada con una limitación de fecha de los últimos 10 años, a fin de presentar la información más actualizada sobre el tema. Los artículos seleccionados incluyeron información referente a las palabras claves utilizadas, como “consultorios odontológicos”, "dosis de radiación”, “rayos X” y “atención ambulatoria”. Se incluyeron estudios observacionales, descriptivos, guías y normativas en radiación odontológica. Se excluyeron las cartas al editor, los artículos de opinión y los editoriales. La información encontrada y analizada corresponde a 41 artículos seleccionados que cumplieron los parámetros establecidos. 

## RESULTADOS Y DISCUSIÓN

### Características generales de los rayos X

Las radiaciones corpusculares y electromagnéticas interactúan con los átomos de cualquier materia, y producen un fenómeno de excitación o ionización ^10^. Los rayos X tienen un menor poder de ionización, pero un gran poder de penetración, contrariamente a las radiaciones corpusculares ^15^.

Para entender las reacciones que pueden ocasionar los rayos X, debemos considerar que, cuando se añade un neutrón positivo a un átomo, se originan isótopos, que son formas de un mismo elemento con diferente número de neutrones en el núcleo ^3^^,^^10^^,^^15^. Los isótopos, a su vez, pueden ser estables, si no emiten radiaciones ionizantes, o radioactivos, si emiten radiaciones ionizantes ^3^^,^^16^. Se encuentran en la naturaleza o pueden ser producidos por el hombre ^3^^,^^15^, lo que determina que las radiaciones sean ionizantes y no ionizantes ^17^.

Por lo descrito anteriormente, existen radiaciones corpusculares ionizantes -como las partículas alfa, beta y neutrónica- y radiaciones electromagnéticas -como los rayos X y gamma-. El hombre ha coexistido a través del tiempo con las radiaciones ionizantes provenientes de fuentes naturales ^16^. El 10% de las exposiciones naturales a las radiaciones ionizantes son de origen natural; por ejemplo, el aire es naturalmente radiactivo, pues contiene radón, un gas inoloro, insípido e invisible, que se produce por la desintegración del uranio ^17^^-^^20^. Se trata de un elemento químico que compone diferentes minerales, como las rocas graníticas. Una partícula alfa convierte el radio (Ra226) en radón 222, el cual se libera en la atmósfera ^21^^-^^23^. La presencia de estos reactivos se da en elementos naturales, la tierra, las rocas y los materiales de construcción, las plantas, los animales y el agua que son consumidos por el hombre ^15^^,^^17^.

Existen pocas posibilidades de disminuir la exposición a la radiactividad natural en la dieta, con una dosis media de 0,29 mSv al año, de los que 0,17 provienen del potasio-40. 

Por otra parte, el 90% de las exposiciones a las radiaciones de los seres humanos tiene fuentes artificiales. Las radiaciones ionizantes generadas por los equipos de rayos X son emitidas por el tiempo que se activa o se realiza el disparo con el equipo y dependen del kilovoltaje, miliamperaje y tiempo de exposición ^6^^,^^18^^,^^24^^-^^25^.

### Efectos biológicos y sus consecuencias en el personal expuesto

Las radiaciones ionizantes pueden provocar consecuencias negativas para la salud ^20^^,^^21^^,^^26^. Los efectos determinísticos representan un umbral de dosis absorbida, definida como una cantidad de energía depositada en un cuerpo por la radiación ionizante Por encima de esta cantidad, se incrementa la gravedad de la lesión y el deterioro de la recuperación. Para los últimos, existe un modelo lineal sin límite, por lo que se puede padecer cáncer o alguna enfermedad heredable por la radiación, sin importar el valor de la dosis ^19^^,^^27^^,^^28^.

Estos efectos generan la muerte o un defecto serio en una célula o grupo de células. Dicho daño tiene que ser continuo para que las lesiones se manifiesten clínicamente de forma importante ^19^^,^^23^^,^^28^. Estos daños también son referidos como efectos cancerígenos y efectos heredables originados por mutaciones celulares somáticas y mutaciones celulares progenitoras. Los estudios experimentales y epidemiológicos establecen que dosis anuales de 100 mSv constituyen dosis de riesgo para el desarrollo de cáncer, sin evidencia directa sobre las enfermedades heredables. Los riesgos de producirse un cáncer están íntimamente relacionados con el daño del ADN de las células ^22^, lo que lleva a las mutaciones en genes-cromosomas, que pueden producirse con dosis bajas (reparación-daño) ^9^^,^^19^.

Los efectos determinísticos se clasifican en tempranos y tardíos. Los efectos tempranos suceden dentro del primer año a la exposición y se asocian con la cantidad de células muertas, la reparación del daño y el recambio de la línea celular, mientras que los efectos tardíos suceden al año de haber recibido la dosis, y se asocian con el daño generado por esta y el deterioro por los mecanismos de reparación ^19^^,^^23^^,^^28^.

La magnitud para medir la radiación es la energía recopilada por unidad de masa, que se denomina dosis absorbida. Esta se refiere a la cantidad de energía que se absorbe en una exposición a las radiaciones ionizantes por unidad de masa irradiada, según la cantidad de energía absorbida por la materia sin que repercutan la clase de radiación y el medio que la absorbe ^2^^,^^3^^,^^29^. La forma en que un tejido es dañado es distinta de acuerdo con las diferentes clases de radiaciones ionizantes. La dosis absorbida con base en el tipo y la calidad de la radiación es conocida como dosis equivalente ^19^^,^^30^^-^^34^.

### Dosis de radiación permitidas para los estudios por imágenes en odontología

Los estudios en radiobiología evidencian que, para el valor de dosis recibida, los daños biológicos son distintos según la radiación a la que fue expuesto. Por ejemplo, la dosis equivalente límite en el cristalino en Argentina se redujo de 150 mSv a 20 mSv al año, lo cual ha hecho que los profesionales deban utilizar anteojos plomados para lograr las dosis señaladas ^28^.

En cuanto a la odontología, los órganos críticos a la radiosensibilidad se encuentran en la cabeza y el cuello, y su grado varía de acuerdo con los órganos, como las glándulas salivales, la piel, la tiroides, el cerebro, la medula ósea, las vías aéreas, el centro de la columna vertebral, el cristalino del ojo y demás ^8^^,^^16^^,^^35^^-^^39^.

Las unidades de radiología pueden ser intraoral, panorámica, teleradiografía, y la tomografía puede ser de haz cónico y multicorte computarizada, empleadas para distintas clases de exámenes de rayos X en radiología dental. El tamaño del área expuesta y la distribución de la dosis de radiación puede variar mucho en la tomografía de haz cónico. Tanto esta como la tomografía multicorte computarizada se están desarrollando velozmente, y serán las más comunes en el futuro cercano ^15^^,^^35^^,^^36^.

A pesar de que la dosis de exposición de la tomografía computarizada de haz cónico es reducida en comparación con la tomografía convencional, es hasta 10 veces mayor que la de la radiografía intraoral y extraoral empleada en odontología ^11^^,^^21^^,^^36^^-^^38^.

Para entender la exposición a radiaciones ionizantes naturales, encontramos que el carbono y el potasio (K-40) emiten 1,2 mSv por año. En el aire encontramos el mismo valor, mientras que los rayos cósmicos emiten una dosis de 0,4 mSv/año; los alimentos, 0,6 mSv/año, y las edificaciones, hasta 0,5 mSv/año ^19^^,^^39^^,^^40^.

Los límites de dosis que se deben manejar para instalaciones y la exposición de los trabajadores se establece con base en límites primarios correspondientes a 100 mSv, promediados en 5 años sin sobrepasar los 20 mSv (1,7 mSv/mes) por año, mientras que el público en general recibe un promedio de 1 mSv/año.

Una dosis efectiva de 1 mSv es el límite de exposición aceptado para un feto durante el tiempo que dure el embarazo. Esta es la norma de exposición para una mujer dentro de un grupo ocupacionalmente expuesto y se considera desde el momento en que ella comunica su estado gestacional.

Dicho límite neonatal es el mismo que se aplica a menores de 10 años por la labilidad de algunos de sus órganos en etapa de formación y maduración. Cuando se evalúa el riesgo para mayores de 10 años, que incluye a los adultos, se calcula que es de 2 a 3 veces menor, por lo que parece aconsejable una restricción de dosis a 3 mSv ^41^.

Por otro lado, el riesgo de exposición para mayores de 60 años es de 3 a 10 veces menos y de 5 a 10 veces inferior para los mayores de 65 años, por lo que se ha calculado que la dosis permitida para ambos grupos es de 15 mSv ^5^^,^^23^^,^^38^.

Las dosis de exposición medidas en dosis efectiva para los estudios por imágenes se generan de acuerdo con los factores de cada equipo, expresados en mSv/uSv. Para tener una idea de la magnitud de lo mencionado, podemos ver una comparación entre valores en estudios odontológicos y las dosis permitidas según organismos internacionales (Tabla 1), así como las dosis recibidas de manera natural. Allí observamos, por ejemplo, que para una radiografía periapical convencional la dosis absorbida aproximada es de 0,0015 mSv (1,5 uSv), valor lejano de los 1,7 mSv permitidos como límite ocupacional mensual, y del ratio establecido de radiación encontrada en la naturaleza.

**Tabla 1:** Datos comparativos para diferentes estudios por imágenes con dosis efectiva para pacientes, para personal ocupacionalmente expuesto y dosis de fuentes naturales

Equipo	Dosis efectiva real para pacientes en estudios por imágenes		Dosis efectiva límite para personal ocupacionalmente expuesto		Dosis efectiva otorgada por la naturaleza
	µSv	µSv	µSv/mes	µSv/año	µSv/año
Radiografía intraoral	1,5	0,0015	1,7	20	2,4
Serie intraoral 14-16 periapicales	21	0,021	1,7	20	2,4
Radiografía panorámica	2,7-24,3	0,0027-2,0243	1,7	20	2,4
Telerradiografía lateral	6	0,006	1,7	20	2,4
CBCT (small FOV)	48-652	0,048-0,652	1,7	20	2,4
CBCT (large FOV)	68-1073	0,068-1,073	1,7	20	2,4
Tomografía médica	280-1410	0,28-1,41	1,7	20	2,4

Por otro lado, existen parámetros de comparación que permiten tener una idea global respecto de la equivalencia aproximada de los estudios en radiología odontológica, en comparación con las emisiones que generan una radiografía de tórax (Tabla 2) y las cantidades de radiación recibidas de fuentes de la naturaleza.

**Tabla 2:** Cuadro comparativo para estudios por imágenes odontológicos y sus equivalencias con radiografía de tórax y dosis de fuentes naturales

Equipo	Dosis (µSv)	No equivalente de radiografías de tórax	Periodo equivalente de radiación natural ambiente
Intraoral	0,002	0,11	1,2 días
Ortopantomografía	0,01	0,5	2,4 días
TC dental*	≤ 0,5 por maxilar	25	10 meses
TC tórax*	8	400	3 años

TC = tomografía computarizada

## CONCLUSIONES

El conocimiento de las dosis absorbidas por los estudios por imágenes en odontología debe estar asociado al uso y la verificación de las condiciones de los equipos de rayos de uso común, pues se debe evitar el empleo innecesario de fuentes de radiación por encima de los parámetros establecidos por las autoridades competentes. Aunque los parámetros de adquisición de imágenes se encuentran por debajo de los límites establecidos, es importante tener en cuenta que las radiaciones ionizantes podrían ocasionar algunas alteraciones no deseadas. El rol del radiólogo oral y maxilofacial debe incluir no solo el apoyo diagnóstico, sino la orientación en el uso de equipos y conocimiento de sus características para un mejor uso.

## Fuente de financiamiento:

Autofinanciado

## References

[1] Kelaranta A, Ekholm M, Toroi P, Kortesniemi M (2016). Radiation exposure to foetus and breasts from dental X-ray examinations: effect of lead shields. Dentomaxillofac Radiol.

[2] Hoogeveen RC, Sanderink GC, Van Der Stelt PF, Berkhout WE (2015). Reducing an already low dental diagnostic X-ray dose: does it make sense? Comparison of three cost-utility analysis methods used to assess two dental dose-reduction measures. Dentomaxillofac Radiol.

[3] Sociedad Española de Protección Radiológica (2007). Las recomendaciones 2007 de la Comisión Internacional de Protección Radiológica.

[4] Olszewski J, Wrzesien M (2017). Evaluation of dental X-ray apparatus in terms of patient exposure to ionizing radiation. Med Pr.

[5] Ubeda C, Nocetti D, Aragón M (2018). Seguridad y protección radiológica en procedimientos imagenológicos dentales. Int J Odontostomat.

[6] Lubis LE, Bayuadi I, Bayhaqi YA, Ardiansyah F, Setiadi AR, Sugandi RD (2019). Radiation dose from dental radiography in Indonesia A five-year survey. Radiat Prot Dosimetry.

[7] Antonio EL, do Nascimento AJ, Soares de Lima AA, Soares MS, Fernandes A Genotoxicity and cytotoxicity of x-rays in children exposed to panoramic radiography. 2016. Revista Paulista de Pediatria.

[8] Felice FD, Di Carlo G, Saccucci M, Tombolini V, Polimeni A (2019). Dental cone beam computed tomography in children: clinical effectiveness and cancer risk due to radiation exposure. Oncology.

[9] Yeh JK, Chen CH (2018). Estimated radiation risk of cancer from dental cone-beam computed tomography imaging in orthodontics patients. BMC Oral Health.

[10] Aanenson JW, Till JE, Grogan HA (2018). Understanding and communicating radiation dose and risk from cone beam computed tomography in dentistry. Journal of Prosthettic Dentistry.

[11] Anissi HD, Geibel MA (2014). Intraoral radiology in general dental practices - a comparison of digital and film-based X-ray systems with regard to radiation protection and dose reduction. Rofo.

[12] Yeung AW, Jacobs R, Bornstein MM (2019). Novel low-dose protocols using cone beam computed tomography in dental medicine a review focusing on indications, limitations, and future possibilities. Clinical Oral Investigations.

[13] Chinem LA, Vilella BD, Mauricio CL, Canevaro LV, Deluiz LF (2016). Digital orthodontic radiographic set versus cone-beam computed tomography an evaluation of the effective dose. Dental Press Journal of Orthodontics.

[14] Nejaim Y, Vasconcelos KF, Roque TG, Meneses LA, Bóscolo FN, Haiter NF (2015). Racionalización de la dosis de radiación. Revista Estomatológica Herediana.

[15] Campillo-Rivera GE, Vázquez-Bañuelos J (2019). Doses in eye lens, thyroid, salivary glands, mammary glands, and gonads, due to radiation scattered in dental orthopantomography. Applied Radiation and Isotopes.

[16] Benchimol D, Koivisto J, Kadesjo N, Shi XQ (2018). Effective dose reduction using collimation function in digital panoramic radiography and possible clinical implications in dentistry. Dentomaxillofac Radiology.

[17] Signorelli L, Patcas R, Peltomaki T, Schatzle M (2016). Radiation dose of cone-beam computed tomography compared to conventional radiographs in orthodontics. Journal of Orofacial Orthopedics;.

[18] Wrzesien M, Olszewski J (2017). Absorbed doses for patients undergoing panoramic radiography, cephalometric radiography and CBCT. Int J Occup Med Environ Health.

[19] Granlund C, Thilander-Klang A, Ylhan B, Lofthag-Hansen S, Ekestubbe A (2016). Absorbed organ and effective doses from digital intra-oral and panoramic radiography applying the ICRP 103 recommendations for effective dose estimations. Br J Radiol.

[20] Radan R (2017). Lowering the radiation dose in dental o ces. J Calif Dent Assoc.

[21] Angelieri F, Yujra VQ, Oshima CTF, Ribeiro DA (2017). Do Dental X-Rays Induce Genotoxicity and Cytotoxicity in Oral Mucosa Cells A Critical Review. Anticancer Res.

[22] Nejaim Y, Silva AI, Brasil DM, Vasconcelos KF, Haiter NF, Boscolo FN (2015). Efficacy of lead foil for reducing doses in the head and neck: a simulation study using digital intraoral systems. Dentomaxillofac Radiol.

[23] Andisco D, Blanco S, Buzzi AE (2014). Dosimetría en radiología. Revista Argentina de Radiología;.

[24] International Organization for Standardization (1996). X and Gamma Reference Radiation for Calibrating Dosemeters and Doserate Meters and for Determining their Response as a Function of Photon Energy, ISO 4037/Part 1: Radiation Characteristics and Production Methods.

[25] International Organization for Standardization (1998). X and Gamma Reference Radiation for Calibrating Dosemeters and Doserate Meters and for Determining their Response as a Function of Photon Energy, ISO 4037/ Part 2: Dosimetry for Radiation Protection over the Energy Ranges 8 keV to 1.3 MeV and from 4 MeV to 9 MeV.

[26] International Organization for Standardization (1998). X and Gamma Reference Radiation for Calibrating Dosemeters and Doserate Meters and for Determining their Response as a Function of Photon Energy, ISO 4037/ Part 3: Calibration of Area and Personal Dosemeters and the Measurement of their Response as a Function of Energy and Angle Incidence.

[27] International Organization for Standardization (2004). X and Gamma Reference Radiation for Calibrating Dosemeters and Doserate Meters and for Determining their Response as a Function of Photon Energy, ISO 4037/ Part 4: Calibration of area and personal dosemeters in low energy X reference radiation fields.

[28] Barros DJ (2011). El radón ¿riesgo para la salud?. Revista de Salud Ambiental.

[29] International Commission on Radiation Protection (2007). The 2007 Recommendations of the International Commission on Radiological Protection ICRP 103. Ann ICRP.

[30] Glosario de Seguridad Tecnológica del OIEA.

[31] Martin CJ (2007). Effective dose: how should it be applied to medical exposures?. Br J Radiol.

[32] Ludlow JB, Davies-Ludlow LE (2008). Patient risk related to common dental radiographic examinations. J Am Dent Assoc,.

[33] Helmrot E, Thilander KA (2010). Methods for monitoring patient dose in dental radiology. Radiation Protection Dosimetry.

[34] Ludlow JB, Ivanovic M (2008). Comparative dosimetry of dental CBCT devices and 64- slice CT for oral and maxillofacial radiology, Oral Surg Oral Med.. Oral Pathol. Oral Radiol Endod;.

[35] Oenning AC, Jacobs R, Pauwels R, Stratis A, Hedesiu M, Salmon B (2017). Cone-beam CT in paediatric dentistry DIMITRA project position statement. Pediatr Radiol.

[36] Pauwels R, Zhang G, Theodorakou C, Walker A, Bosmans H, Jacobs R, Bogaerts R, Horner K, SEDENTEXCT Project Consortium (2014). Effective radiation dose and eye lens dose in dental cone beam CT effect of field of view and angle of rotation. Br J Radiol.

[37] Hidalgo RJ, Theodorakou C, Carmichael F, Murray B, Payne M, Horner K (2014). Use of cone beam CT in children and young people in three United Kingdom dental hospitals. Int. J. Paediatr Dent;.

[38] Ludlow JB, Timothy R, Walker C, Hunter R, Benavides E, Samuelson DB (2015). Effective dose of dental CBCT-a meta-analysis of published data and additional data for nine CBCT units. Dentomaxillofac Radiol.

[39] Raelson CA, Kanal KM, Vavilala MS, Rivara FP, Kim LJ, Stewart BK (2009). Radiation dose and excess risk of cancer in children undergoing neuroangiography. Am J Roentgenol.

[40] Roberts JA, Drage NA, Davies J, Thomas DW (2009). Effective dose from cone beam CT examinations in dentistry. Br J Radiol,.

[41] Sonawane AU, Sunil KJ, Singh M, Pradhan AS (2011). Suggested diagnostic reference levels for pediatric X-ray examinations in India. Radiat Prot Dosim.

